# Gender Differences in Pain Perception and Functional Ability in Subjects with Knee Osteoarthritis

**DOI:** 10.5402/2012/413105

**Published:** 2012-08-30

**Authors:** M. Elboim-Gabyzon, N. Rozen, Y. Laufer

**Affiliations:** ^1^Physical Therapy Department, Faculty of Social Welfare & Health Sciences, University of Haifa, Haifa 31905, Israel; ^2^Emek Medical Center, Afula 18101, Israel

## Abstract

*Background*. There is no consensus regarding gender-related differences in pain intensity and functional abilities among patients with knee osteoarthritis (OA). *Objective*. Determine gender-related differences in pain intensity and functional ability among subjects with knee OA, as assessed by a self-report questionnaire and by performance-based tests. *Methods*. Sixty-three subjects with symptomatic knee pain due to OA were included in this study. The outcome measures were self-reported knee pain intensity and physical function (WOMAC), as well as three performance-based functional assessments: time up and go test, a 10-meter walk test, and stair negotiation. Independent sample *t*-tests were performed to determine gender differences. Level of significance was set at *P* ≤ 0.05. *Results*. Female subjects reported higher levels of knee pain and lower functional performance. In contrast, no significant gender-related differences were determined in any of the performance-based measures. *Conclusion*. The results indicate that the two types of functional ability measures may address different constructs of functional ability. Self-reported ability, particularly in the female subjects, may be influenced by psychological aspects associated with chronic pain. Rehabilitation programs should consider the underlying mechanisms of the patients' performance limitations in order to address the specific needs of each individual patient.

## 1. Introduction 

Knee osteoarthritis (OA) is one of the most common forms of arthritis in Western populations [1]. One of the known predisposing factors of knee OA is female gender, leading to a higher prevalence of symptomatic knee OA in female [1]. Pain is the most common impairment associated with knee OA [1], and knee OA is one of the main causes of long-term disability in people over 65, leading to moderate to severe limitations in participation and a reduction in quality of life [1]. 

Gender differences in the clinical status of subjects with chronic pain are likely to affect the patients' treatment-seeking behavior, the evaluation and treatment approach, and the responsiveness to treatment [2]. In spite of the higher prevalence of knee OA among women, gender-related differences among patients with knee OA have received little attention. Thus, there is no consensus in the literature on gender-related differences in pain level intensity reported by these patients [3, 4], and reports regarding gender-related differences in functional abilities are inconsistent [5, 6]. However, most of these studies have focused on self-report measures alone [7]. A comprehensive assessment of the functional abilities of patients with knee OA should include not only self-report measures, but performance measures as well [7, 8]. Therefore, it is necessary to further elucidate gender-related differences in patients with knee OA. The aim of this study is to characterize gender differences in subjects with knee OA regarding pain intensity and functional ability, as assessed by a self-report questionnaire as well as by actual performance tests.

## 2. Study Methodology

### 2.1. Subjects

Subjects were 63 patients, including 52 females and 11 males, who were diagnosed with knee OA and referred to physical therapy by a general practitioner/orthopedist or rheumatologist. Inclusion criteria were (1) in accordance with the classification of the American College of Rheumatology, subjects presented with at least three of the following: (a) age above 50, (b) morning stiffness less than 30 minutes long, (c) crepitus, (d) bony tenderness, (e) bony enlargement and (f) no palpable warmth; (2) knee pain for at least three months, with pain presenting at least three days a week during the last month; (3) ability to ambulate independently for at least 10 meters (with or without assistive devices); (4) ability to follow instructions, communicate and cooperate; (5) ability to complete the WOMAC questionnaire in Hebrew; and (6) grade ≥II radiographic evidence of knee OA in at least one knee compartment according to the Kelgren and Lawrence classification. The radiographs, taken within three months of assessment, were graded by an orthopedist with expertise in knee OA. 

Exclusion criteria were (1) history of cardiovascular, neurological, or orthopedic problems that could affect functional performance (such as stroke, uncontrolled angina pectoris, or prior leg fracture), (2) previous knee surgery, (3) injections to the knee joint during the previous six months, or (4) change in pain medication in the last month. 

The Human Subjects Review Committee at the Emek Medical Center, Klalit Health Services approved the study. All subjects signed an informed consent form prior to assessment.

### 2.2. Assessments

The subjects completed the Western Ontario and McMaster Universities Osteoarthritis Index (WOMAC), which is a disease-specific self-report questionnaire designed to examine relevant symptoms in patients with OA of the knee and/or hip. It includes 24 items, with 5 items evaluating knee pain, 2 items evaluating stiffness, and 17 items evaluating physical function. Each item is evaluated on a 10 cm visual analogue scale (VAS), with 0 representing no pain and 10 cm representing the most severe pain. Higher scores indicate higher levels of pain and stiffness and lower functional status. The total WOMAC scores and the scores for each category were used for analysis. The reliability and validity of the WOMAC have been previously established. The Hebrew version has also been shown to be a reliable and valid instrument for evaluating the severity of knee OA in Israeli patients [9].

The participants were instructed to perform the following three performance-based assessments at a self-selected comfortable pace. Time to complete the tasks was measured with a digital stopwatch. Subjects were allowed to rest between tests for as long as needed, with a minimum rest time of five minutes between tests. The tests were implemented in the same order by all patients, as presented herein.

#### 2.2.1. The Ten-Minute Walk Test (10 MW)

A 14-meter indoor course was delineated with masking tape markers placed at 0 meters, 2 meters, 12 meters, and 14 meters. The actual course length was 10 m (between the 2 and 12 meter marks) in order to minimize the effects of acceleration and deceleration. The mean time of two trials was averaged. Reliability and validity have been established for the 10 MW test among people with knee OA. 

#### 2.2.2. The Timed Up and Go Test (TUG)

Subjects were requested to rise from a seated position in an arm chair (seat height 46 cm), walk three meters, turn around, and return to a seated position without assistance. The subjects were permitted to use the arms of the chair to assist with standing and returning to a seated position. The test was performed twice, and the times were averaged. The TUG has been used extensively to examine functional outcomes in patients with patients with knee OA or after total knee arthroplasty (TKA) and has been proven to be a valid and reliable test. 

#### 2.2.3. Stair Test

Participants ascended, turned around, and then descended eight standardized 17 cm high stairs. The subjects were allowed to use the handrails as necessary. This test has been widely accepted as a valid and reliable functional measure in knee OA. 

### 2.3. Statistical Analysis

Descriptive statistics (mean, standard deviation, and range) were calculated for all outcome measures. Independent samples *t*-tests were performed to determine gender differences. Level of significance was set at *P* ≤ 0.05. Statistical analyses were performed using JMP (SAS Institute, Cary, NC).

## 3. Results

Sixty-three subjects (52 females) complying with the inclusion/exclusion criteria referred to the physical therapy department between September 2008 and December 2009 were included. Clinical characteristics of the subjects by gender are presented in Table 1. 

Subjects' test results, by gender, are presented in Table 2. Analysis revealed significant gender differences for the WOMAC questionnaire (total score). The female reported higher levels of knee pain and greater stiffness in comparison to the male. Additionally, the female reported lower functional performance. In contrast, no significant gender differences were determined for any of the three performance-based measures.

## 4. Discussion

The aim of this study was to determine gender differences in terms of knee pain and functional ability in patients with symptomatic knee OA. Female represented 87% of the current sample, in line with the higher prevalence of knee OA among female reported in the literature [1]. The female subjects in the present study reported a higher intensity of knee pain in comparison with the male subjects, which is consistent with some previous studies [4, 10]. The differences in the reports may be related to differences in the background characteristics of the subjects (e.g., OA severity, duration, and age). 

The female subjects also reported significantly lower functional abilities in comparison to the male subjects, which is consistent with most previous reports [3, 4]. This difference in self-reported ability is in contrast to the lack of significant gender-related differences observed in actual functional performance, as assessed by the 10 MW test, the TUG test, and the stair negotiation test. This is of particular interest, as a large proportion of the items included in the self-report WOMAC are related to the same functional performance tests that were actually conducted (e.g., walking, sitting up and down, stair negotiation).

A possible explanation for the current discrepancy between genders in self-reported function and objective functional tests may be related to the fact that these two types of measures examine different constructs of functional ability. While performance-based tests measure the actual ability to perform a physical task, the WOMAC, as a self-report assessment, measures the individual perceptions of one's ability to perform the task [8]. 

Previous studies indicate that self-report measures are strongly related to psychological factors, such as pain catastrophizing, self-efficacy, and depression [8], and that such psychological factors may be gender related [11]. Pain catastrophizing is defined as the patient's belief that the pain will progress and that the patient is helpless to deal with it. Studies have demonstrated that pain catastrophizing is influenced by gender in subjects with knee OA [4]. Keefe et al. (2000) [4] also determined that when the pain catastrophization factor in these patients was isolated, there were no significant gender-related differences in pain intensity or in self-reported functional ability. 

Self-efficacy refers to the individual's cognitive belief in his/her own ability, which is based on previous performance or behavior, observations of others modeling the behavior, and encouragement from others when performing the behavior [12]. Self-efficacy has also been shown to be gender related in people with various pathologies (e.g., cancer, chronic obstructive lung disease) [10]. Gaines et al. (2004) [13] demonstrated that stronger self-efficacy beliefs were significantly associated with higher self-reported functional ability in female subjects with knee OA, but not in male subjects. Finally, Tsai (2007) [14] found that females with knee OA had a greater depressive tendency than their male counterparts. Thus, psychological factors may be an additional variable affecting the observed gender-related difference in self-reported functional ability. 

## 5. Summary

Gender differences were observed in the current study only for pain intensity and self-reported functional ability. In contrast, no gender differences were found in actual functional performance. This indicates that these two types of measures may address different constructs of functional ability and that self-reported ability, particularly in the female subjects, may be influenced by psychological aspects associated with chronic pain. It is, therefore, necessary to determine the underlying mechanism affecting the differences observed in the self-reported measures. Understanding these constructs will help to develop appropriate rehabilitation programs that address the specific needs of these patients.

## Figures and Tables

**Table 1:** Clinical characteristics (mean ± SD) of subjects by gender and comparison between genders.

	Male (*n* = 11)	Female (*n* = 52)	*P* value
Age (years)	75 ± 8.4	66.8 ± 7	0.001
Weight (Kg)	87.6 ± 14.1	77.7 ± 15.5	0.05
Height (cm)	172.6 ± 2.5	158.4 ± 5.4	0.001
BMI (Weight/Height^2^)	29.4 ± 4.3	30.9 ± 5.7	0.43
Knee OA duration (years)	2.7 ± 4.1	4.7 ± 5.7	0.29

Kg: kilograms, cm: centimeter; BMI: body mass index.

**Table 2:** Functional ability variables (mean ± SD) by gender and comparison between genders.

	Male (*n* = 11)	Female (*n* = 52)	*P* value
WOMAC			
Pain (0–50)	14.8 ± 7.6	23.6 ± 10.6	*P* = 0.01**
Stiffness (0–20)	4.1 ± 4	8 ± 5.6	*P* = 0.04*
ADL (0–170)	43.4 ± 28.6	77.8 ± 36.1	*P* = 0.004**

Total (0–240)	62.3 ± 38.6	109.8 ± 48.3	*P* = 0.003**

10 MW (sec)	9.5 ± 2	10.8 ± 2.9	*P* = 0.14
Stair negotiation (sec)	16.1 ± 8.7	19.4 ± 8.4	*P* = 0.25
TUG (sec)	11.1 ± 3.1	12.9 ± 3.8	*P* = 0.14

*Denotes statistical significance *P* ≤ 0.05, **Denotes statistical significance *P* ≤ 0.01.

WOMAC: Western Ontario and McMaster Universities Osteoarthritis Index; ADL: activities of daily living; 10 MW: 10-meter walk test; sec: seconds; TUG: timed up and go test.
